# Stabilized Cellulase in Chitosan–Polyvinyl Alcohol Biopolymer Beads for Sustainable Enzymatic Deinking of Recycled Paper

**DOI:** 10.1002/open.202500326

**Published:** 2025-10-08

**Authors:** Parisa Chakeri, Ghasem Mohammadi‐Nejad, Ghasem Hosseini Salekdeh, Shohreh Ariaeenejad, Azadeh Lohrasbi‐Nejad

**Affiliations:** ^1^ Department of Agricultural Biotechnology Shahid Bahonar University of Kerman Kerman +98 Iran; ^2^ Institute of Plant Production (IPP) Afzalipour Research Institute (ARI) Shahid Bahonar University of Kerman Kerman +98 Iran; ^3^ Department of Molecular Sciences Macquarie University Sydney 2109 NSW Australia; ^4^ Department of Systems and Synthetic Biology Agricultural Research Education and Extension Organization (AREEO) Agricultural Biotechnology Research Institute of Iran (ABRII) Karaj +98 Iran; ^5^ Department of System Biotechnology Institute of Plant Production (IPP) Afzalipour Research Institute (ARI) Shahid Bahonar University of Kerman Kerman +98 Iran

**Keywords:** biodeinking, cellulase immobilization, chitosan–polyvinyl alcohol beads, cross‐linker, thermostable enzyme

## Abstract

Developing stable and reusable biocatalysts is crucial for improving the sustainability of industrial processes, including enzymatic deinking. In this article, cellulase is immobilized onto chitosan–polyvinyl alcohol (Cs/PVA/Ga) biopolymer beads using glutaraldehyde cross‐linking, creating a durable and recyclable catalytic system. Scanning electron microscopy revealed a bead‐like structure, and fourier transform infrared spectroscopy spectra confirmed successful enzyme incorporation without compromising the polymer's integrity. Immobilization shifted the optimal activity of cellulase from pH 5 to pH 8 and raised the temperature optimum from 50 °C to between 60 and 70 °C, indicating improved catalytic stability. Kinetic studies showed a decrease in Km from 0.75 mM for the free enzyme to 0.4mM for the immobilized form, suggesting increased substrate affinity. Thermal stability tests revealed cellulase@Cs/PVA/Ga maintained over 83% of its activity at 80 °C for 60 min, compared to only 50% for the free enzyme. The immobilized cellulase demonstrated 90% activity retention after seven reuse cycles. Biodeinking experiments with recycled pulp evidenced optimal cellulose and hemicellulose retention with a 5% enzyme dosage, while effluent analysis showed enhanced removal of ink residues with the immobilized enzyme, highlighting the ecoefficient potential of this approach for sustainable paper recycling.

## Graphical abstract

A stable and reusable biocatalyst, cellulase@Cs/PVA/Ga, is engineered by immobilizing cellulase onto glutaraldehyde‐activated chitosan (Cs) and coating it with polyvinyl alcohol. This composite system enhances enzymatic stability and efficiency for the sustainable deinking of recycled newspaper, offering a green alternative to harsh chemical treatments.

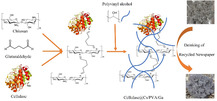

## 1 Introduction

Recycling paper mitigates deforestation and environmental damage while conserving vital resources such as water, oil, and natural gas.^[^ ^1^ ^]^ Recycling organic paper waste, including newspaper, photocopies, and laser prints, involves repulping to create a fiber slurry and deinking to remove ink.^[^ ^2^ ^]^ Ink residues complicate deinking and reduce paper brightness, necessitating effective deinking methods. Because traditional chemical methods generate pollutants and increase wastewater treatment expenses, greener alternatives are urgently needed.^[^ ^3^ ^,^ ^4^ ^]^ Lignocellulosic enzymes play a critical role in enhancing the efficiency and sustainability of the pulp and paper industry by modifying fibers, lowering energy consumption, and improving product quality.^[^ ^5^ ^]^

Cellulases are used in various processes in the pulp and paper industry, from improving the drain ability of secondary fibers to enzymatic deinking of recycled papers.^[^ ^6^ ^]^ Cellulases are vital for enzymatic deinking as they facilitate ink removal through the breakdown of cellulose fibers, leading to higher‐quality recycled materials.^[^ ^7^ ^,^ ^8^ ^]^ Enzymes are important for making kraft pulp brighter and more stable.^[^ ^9^ ^]^

However, using them can be challenging because environmental changes, such as temperature and pH levels, can affect them. These changes can reduce how well they work and shorten their lifespan. Environmental sensitivity, particularly to temperature and pH, limits the industrial application of free enzymes due to reduced catalytic efficiency and lifespan. Strict reaction condition requirements complicate scale‐up and necessitate protection against shear and chemical inhibitors.^[^ ^10^, ^11^, ^12^, ^13^ ^–^ ^14^ ^]^

To overcome limitations in industrial applications, advanced enzyme immobilization techniques and robust formulations are needed to improve cellulase stability, activity retention, and reusability. While cellulase can be immobilized through physical adsorption,^[^ ^15^ ^,^ ^16^ ^]^ covalent bonding,^[^ ^17^, ^18^, ^19^ ^–^ ^20^ ^]^ or encapsulation,^[^ ^21^ ^,^ ^22^ ^]^ covalent bonding offers distinct advantages. Linking cellulase to chitosan with glutaraldehyde as a cross‐linker, forming stable covalent bonds significantly enhances enzyme stability and minimizes leaching.^[^ ^13^ ^]^

Chitosan‐based supports provide a suitable environment for enzyme immobilization and have biocompatibility, nontoxicity, and ease of modification.^[^ ^20^ ^]^ In covalent bonding, bifunctional linkers are utilized to connect both the support and the enzyme, including diamines, diacids, and dialdehydes.^[^ ^23^ ^]^ Glutaraldehyde's reactivity with primary amino groups and other functional groups, such as phenol, thiol, and imidazole, makes it effective for enzyme immobilization.^[^ ^24^, ^25^ ^–^ ^26^ ^]^ Immobilizing cellulase on chitosan improves performance and supports sustainable practices. Its biodegradability and reduced chemical usage minimize the environmental impact of paper recycling, aligning with global sustainability efforts.

This study focused on the simultaneous synthesis of chitosan beads and the immobilization of cellulase using glutaraldehyde. Various instrumentation techniques validated the immobilization of cellulase (cellulase@Cs/PVA/Ga), and we examined the optimal conditions and stability of the immobilized enzyme activity. We also explored the potential of cellulase@Cs/PVA/Ga beads for biodeinking recycled paper.

## 2 Results and Discussion

### 2.1 Morphology Studying

**Figure 1:**
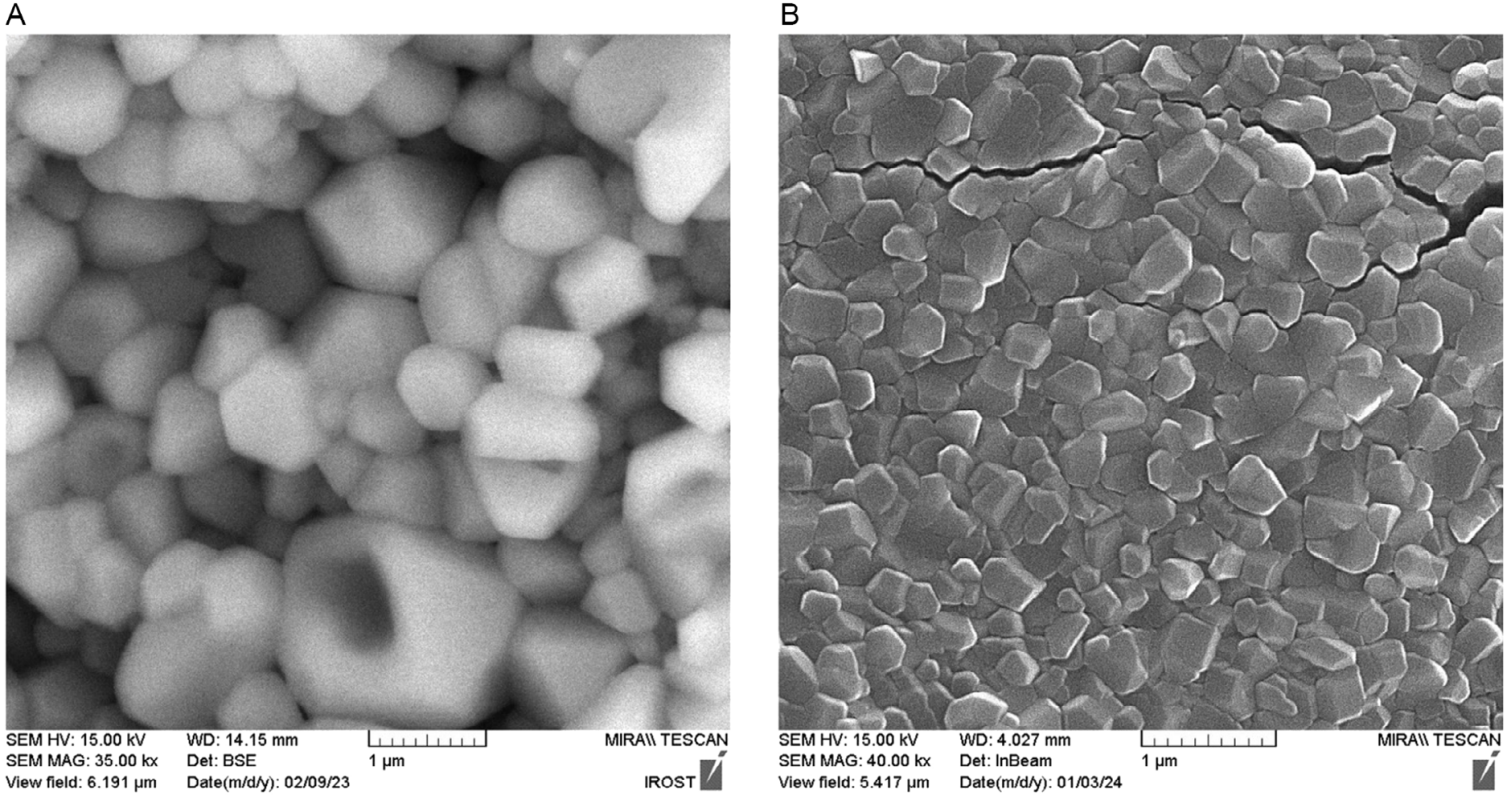
Morphology of synthesized A) Cs/PVA/Ga and B) cellulase@Cs/PVA/Ga beads.

The scanning electron microscopy indicated the surface morphology of bare Cs/PVA/Ga and cellulase@Cs/PVA/Ga (**Figure** **1**A, B). Both types of cross‐linked Cs/PVA/Ga display a bead structure, consistent with the findings of Li et al. and Galan et al.^[^ ^27^ ^,^ ^28^ ^]^ During synthesis, the chemical cross‐linking between chitosan and cellulase did not impact bead formation. The particle size analysis via ImageJ software showed that Cs/PVA/Ga had a mean diameter of ≈495 nm, while the cellulase@Cs/PVA/Ga had a mean diameter of about 350 nm. The size distribution for the control beads ranged from 0.29 to 0.89 µm, and for the cellulase@Cs/PVA/Ga beads, it ranged from 0.21 to 0.65 µm. The standard deviation was around 0.16 µm for Cs/PVA/Ga and 0.09 µm for cellulase@Cs/PVA/Ga, indicating a relatively narrow spread of particle sizes for both types.

Incorporating glutaraldehyde into PVA/CS blends enhances the polymers’ mechanical and physical properties of the polymers. This enhancement is attributed to stronger intermolecular interactions, including covalent Schiff base linkages with the amino groups of chitosan, acetal bridges with the hydroxyl groups of PVA, and reorganization of hydrogen bonding. These interactions contribute to a more robust bead structure, improved water stability, reduced swelling, and greater thermal resistance.^[^ ^29^ ^,^ ^30^ ^]^

The morphology of chitosan/PVA blends is influenced by solution viscosity and manufacturing techniques. For instance, electrospinning chitosan/PVA solutions can produce smooth, defect‐free nanofibers without bead formation.^[^ ^31^ ^]^ Conversely, low‐viscosity solutions with lower chitosan concentrations are likely to form beads. Therefore, optimizing viscosity is crucial for minimizing nanofiber occurrence and achieving uniform beads.^[^ ^31^ ^,^ ^32^ ^]^ According to a published review paper, many factors, like electrospinning voltage, environmental conditions, and electrospinning solution characteristics, including pH, viscosity, chitosan molecular weight, and polymer/solvent ratios, are crucial in determining the properties of the nanofiber or bead structure.^[^ ^33^ ^]^ For instance, the voltage affects both the diameter of the nanofibers and the surface structure of the nanofiber mat; a higher feed rate results in thicker fibers, low viscosity results in bead formation, and a decrease in chitosan's molecular weight also causes beads to form.^[^ ^33^ ^]^

### 2.2 Fourier Transform Infrared Spectroscopy (FTIR) Analysis

**Figure 2:**
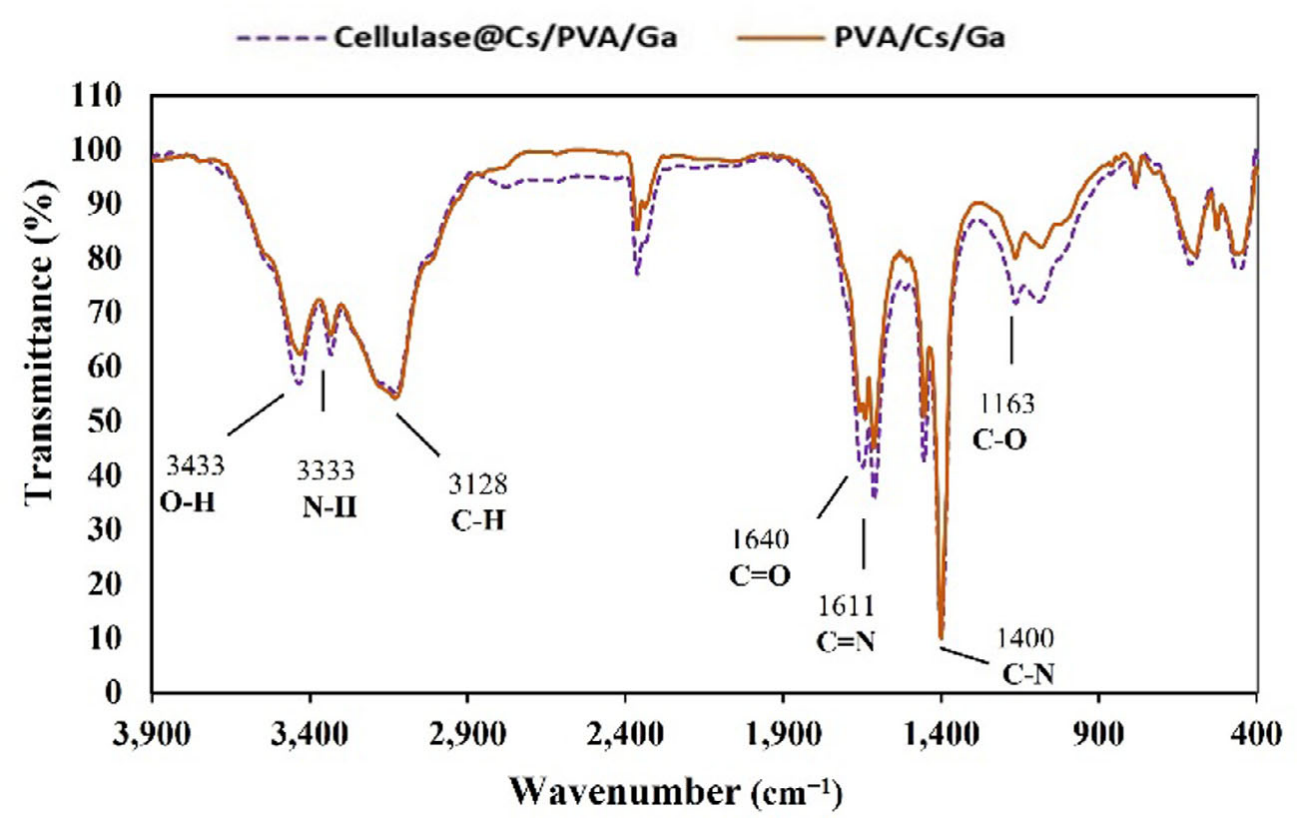
The FTIR spectrum of Cs/PVA/Ga and cellulase@Cs/PVA/Ga.

The FTIR spectrum of cellulase@Cs/PVA/Ga and the control group Cs/PVA/Ga (**Figure** **2**) displays bands at 3433 cm^−1^ for —OH stretching^[^ ^34^ ^]^ and a distinct peak at 3333 cm^−1^ for N—H stretching vibration.^[^ ^27^ ^,^ ^35^ ^]^

The broad O—H/N—H bands suggest a hydrogen‐bonding network that functions as reversible cross‐links around the GA‐fixed enzyme. These interactions help preserve enzyme conformation and hydration, reducing thermal and operational denaturation. A similar behavior was described by Cotugno et al. in epoxy systems, where hydrogen‐bond networks stabilized the matrix under sorption cycles.^[^ ^36^ ^]^ In the case of cellulase@Cs/PVA/Ga, these interactions are expected to promote long‐term enzymatic stability. Although O—H and N—H groups are typically associated with increased hydrophilicity and swelling, their involvement in intermolecular hydrogen bonding (as evidenced by broadened FTIR bands) helps restrict excessive swelling.^[^ ^37^ ^]^ This controlled water uptake enables sufficient substrate diffusion while preventing bead loosening or enzyme leaching, thus helping to preserve enzyme activity.

The peak at 3100 cm^−1^ is attributed to the C—H stretching of the aromatic structure.^[^ ^34^ ^]^ Bands at 1163 and 1400 cm^−1^ correspond to C—O and C—N stretching vibrations, respectively.^[^ ^27^ ^,^ ^38^ ^,^ ^39^ ^]^ The band at 1640 cm^−1^ is attributed to C=O stretching in the amide group of chitosan, formed by partial deacetylation of chitin during chitosan preparation.^[^ ^40^ ^]^ The C=N imine bond, resulting from glutaraldehyde cross‐linking with chitosan amino groups, appears around 1611 cm^−1^,^[^ ^40^ ^,^ ^41^ ^]^ confirming imine bond formation in the enzyme‐containing chitosan beads. Based on the previous study, increased peak intensity at this region indicates a higher number of C=N groups interacting with glutaraldehyde.^[^ ^40^ ^]^ In FTIR spectroscopy, greater intensity of a specific peak typically reflects a higher concentration of the corresponding functional groups, which may suggest new interactions or cross‐linking events.^[^ ^42^ ^]^ For example, Pragya et al. reported that FTIR band intensity was directly related to covalent and ionic cross‐linking in alginate–acrylamide hydrogels.^[^ ^43^ ^]^ Thus, the observed increase near 1611 cm^−1^ in our study suggests an enhanced imine bond formation. The spectral analysis of cellulase@Cs/PVA/Ga showed no significant differences from Cs/PVA/Ga, likely due to similar cross‐linking mechanisms in both systems.

### 2.3 Identification of Optimum pH and Temperature

**Figure 3:**
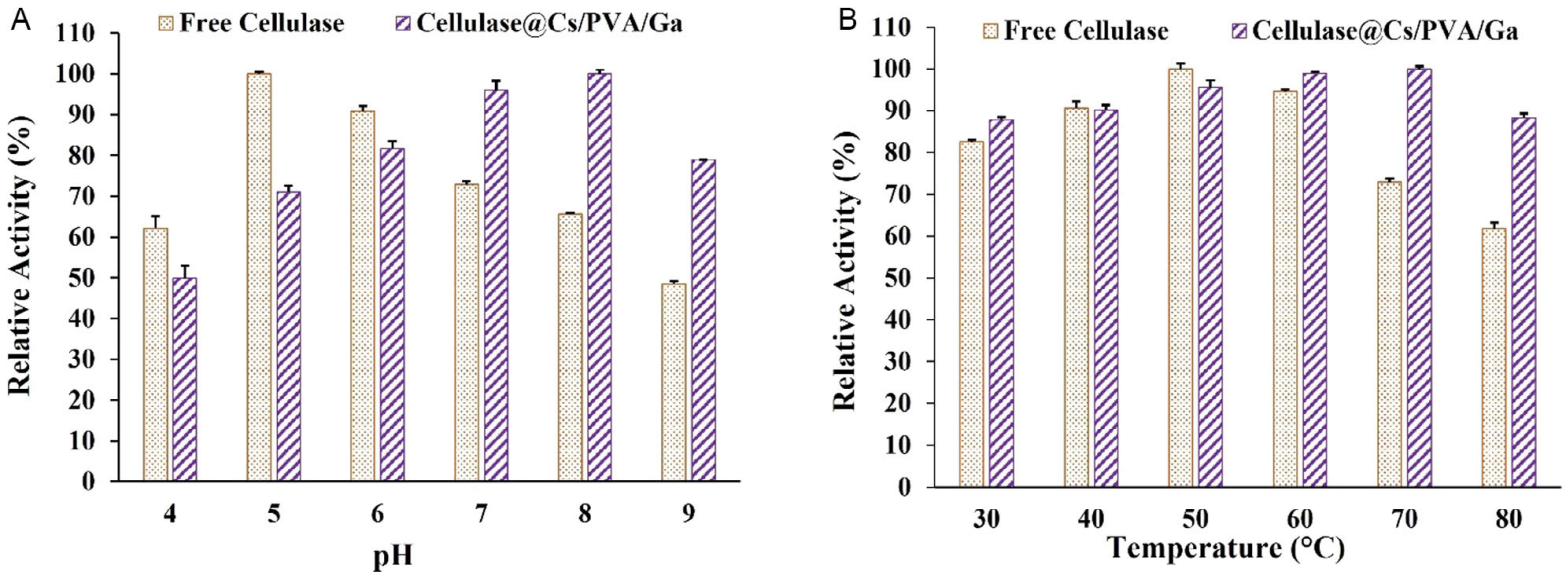
Optimum A) pH and B) temperature of free and immobilized cellulase.

The optimal pH for the enzymes was analyzed from 4 to 9 (**Figure** **3**A). Free enzymes showed peak activity at pH 5, declining in the 7–9 range, while immobilized enzymes exhibited maximum activity (100%) at pH 8, where free enzymes only reached 65%. The optimal pH for immobilized cellulase may vary based on the method and reaction conditions. Coimmobilizing the enzyme during chitosan support synthesis likely enhanced its optimal pH.

This result contrasts with previous studies on cellulase immobilized on various chitosan supports (**Table** **1**). Many studies suggest free and immobilized cellulase peak activity at pH 5 (Table 1). However, some research indicates that the pH for immobilized enzymes can shift to a higher range.^[^ ^44^, ^45^ ^–^ ^46^ ^]^ In a study using melamine–glutaraldehyde dendrimer‐functionalized magnetic nanoparticles (not chitosan‐based), the optimal pH for immobilized cellulase changed from 5 to 8 postimmobilization.^[^ ^47^ ^]^

**Table 1:** Comparison of optimum pH and temperature for free and immobilized cellulase on known chitosan supports.

Type of support	Optimum pH		Optimum temperature [°C]		Reference
	Free enzyme	Immobilized enzyme	Free enzyme	Immobilized enzyme	
Polyvinyl alcohol‐coated chitosan beads	4	7	40	40	[44]
Chitosan‐coated magnetic nanoparticles modified with α‐ketoglutaric acid	3–4	4–7	40	50	[45]
Magnetic chitosan nanoparticles	5	5	50	50	[59]
Magnetic chitosan microspheres	5	5	50	50	[67]
Fe_3_O_4_/GO/CS	5	5	50	50	[68]
Sodium alginate (SA)‐polyethylene glycol (PEG)‐chitosan (CS)	4	5	50	70	[69]
Fe_3_O_4_/GO/CS nanocomposites	6	8	60	80	[49]
Chitosan functionalized magnetic nanoparticles	5	6			[70]
Cs@Fe_3_O_4_	5	7	60	80	[46]
Cs/PVA/Ga	5	8	50	80	This study

Their findings noted that free enzymes had the best activity at acidic pH, while immobilized enzymes performed poorly in acidic conditions but excelled in alkaline environments. They attributed this phenomenon to the susceptibility of Schiff base degradation into aldehyde and amine during synthesis in acidic conditions, which may negatively affect enzyme activity and interfere with enzyme‐carrier interactions.^[^ ^47^ ^]^

Our research investigated enzyme activity across a 30–80 °C temperature range. As illustrated in Figure 3B, the free cellulase reached peak activity at 50 °C, whereas the cellulase@Cs/PVA/Ga performed best in the range of 60–70 °C. Cellulase@Cs/PVA/Ga significantly demonstrated greater activity than the unbound enzyme at elevated temperatures, consistent with earlier results (Table 1). A prior study on the immobilization of mannanase onto chitosan suggested that the elevated optimal temperature of the immobilized enzyme may stem from a synergistic effect with the chitosan beads.^[^ ^48^ ^]^ The immobilization matrix likely creates a favorable microenvironment at the enzyme's active site, promoting substrate interaction while maintaining the enzyme's structure and function at elevated temperatures. However, the extent of this effect can vary based on the specific support materials used and the enzyme‐support interactions.^[^ ^48^ ^]^

### 2.4 Kinetic Studies of Free and Immobilized Cellulase

Kinetic parameters were established for both free and immobilized cellulase using substrate concentrations of 0.1–2.5 mg mL^−1^. Free cellulase had a Km of 0.75 mM and a Vmax of 25.5 µmol min^−1 ^mg^−1^, while cellulase@Cs/PVA/Ga had a Km of 0.4 mM and a Vmax of 18.7 µmol min^−1 ^mg^−1^.

The observed decrease in Vmax and Km after immobilization on chitosan beads indicates an improved affinity for substrate binding. This is consistent with previous cellulase research associated with chitosan‐coated nanoparticles.^[^ ^46^ ^,^ ^49^ ^]^ This improvement may arise from diminished steric hindrance,^[^ ^46^ ^]^ which can obstruct the interaction between the enzyme and substrate. In contrast to the immobilized variant, the higher Km of the free cellulase reinforces the idea of a weaker binding to the substrate.

### 2.5 Determining Thermostability and Storage Stability

**Figure 4:**
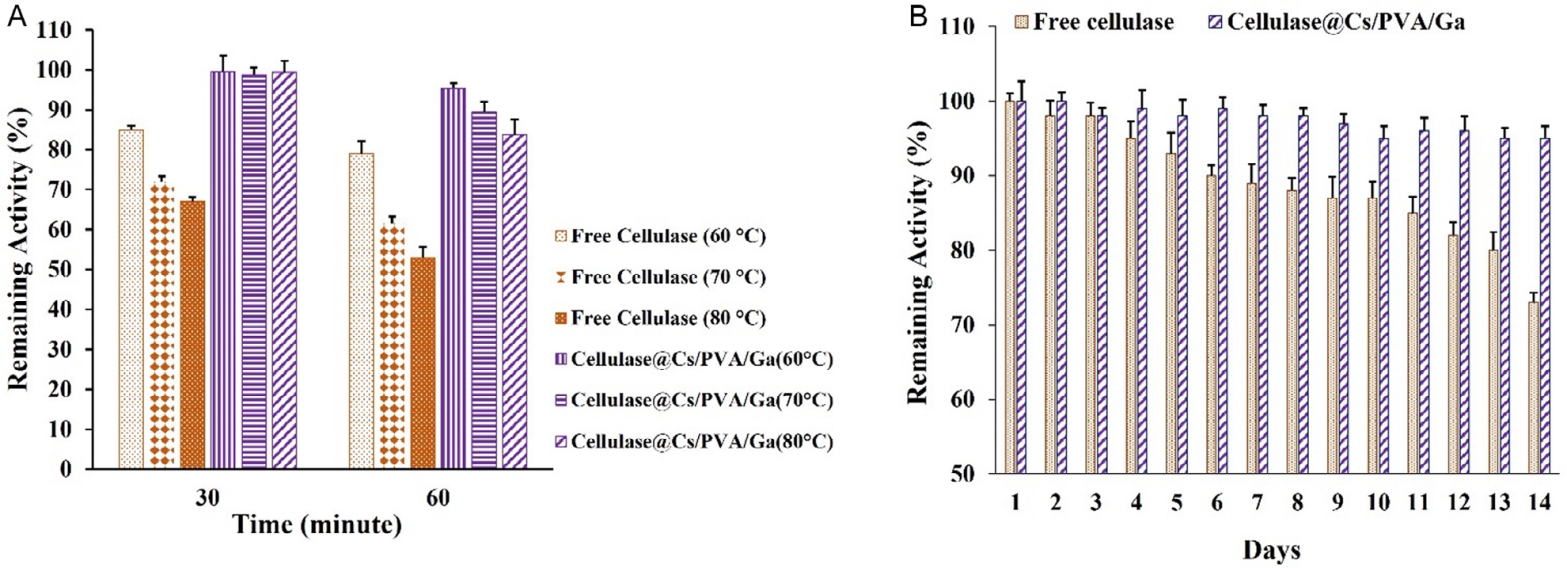
The stabilities of free and immobilized cellulase. A) Thermal stability and B) storage stability.

The thermostability of free and immobilized cellulase was also studied every 30 min at 60, 70, and 80 °C. The activity of free cellulase decreased over time at all temperatures. Consequently, the residual activity of bare cellulase reached 79%, 60%, and 50% at 60, 70, and 80 °C, respectively, after 60 min (**Figure** **4**). However, it seems that immobilization enhanced the stability of the enzyme. The remaining cellulase@Cs/PVA/Ga activity was 99% during the thermal incubation (30 min) at all temperatures. Gradually, over time, the level of immobilized activity decreased to 95%, 89%, and 83% at 60, 70, and 80 °C, respectively, after 60 min. These results indicate that covalent immobilization significantly enhances cellulase thermostability, making the immobilized enzyme more suitable for industrial applications than free cellulase.

Based on Figure 4B, the activity level of free cellulase dropped to 73% of its initial activity after 14 days, whereas, the activity of the immobilized enzyme decreased only to 95%. Free cellulase exhibited a trend of inactivation at 4 °C, while immobilized cellulase retained high activity under the same conditions.

### 2.6 Thermal Inactivation and Thermodynamic Parameters

The study of enzyme thermal inactivation at elevated temperatures aimed to enhance understanding of the relationship between enzyme structure and function. The activation energy (Ea) was calculated from the slope of the Arrhenius plot for the thermal inactivation of both immobilized and free enzymes (**Table** **2**). The results showed Ea values of 55.89 kJ mol^−1^ for free cellulase and 47.95 kJ mol^−1^ for cellulase@Cs/PVA/Ga. A higher Ea indicates greater sensitivity to temperature changes,^[^ ^50^ ^]^ suggesting that free cellulase has a more thermally sensitive conformation than its immobilized counterpart. An important thermodynamic parameter is Δ*G*, which reflects the energy required to surpass the activation energy barrier.^[^ ^51^ ^]^ The ΔG values for cellulase@Cs/PVA/Ga were higher than those for the free enzyme at various temperatures, indicating that immobilized cellulase requires more energy for thermal inactivation.^[^ ^52^ ^]^ Based on previous studies, the nanoparticle matrix enhances catalytic activity by lowering the activation energy (Ea) through increased rigidity, multipoint covalent binding, and stabilization of the enzyme's microenvironment. Consequently, ΔG for thermal inactivation increases, as the enzyme's conformation becomes more constrained and its unfolding entropy is reduced.^[^ ^53^ ^,^ ^54^ ^]^ The values o*f* Δ*H and* Δ*S* related to changes in enthalpy and entropy affect the conformational changes and interactions of proteins, such as hydrogen bonding, Van der Waals forces, and electrostatic attractions. A positive Δ*S* indicates significant protein aggregation during thermal inactivation, while negative values suggest conformational changes. ΔS highlights the enzyme's conformational changes and flexibility in structure.^[^ ^51^ ^]^

**Table 2:** Thermodynamic parameters of thermal inactivation of free and immobilized cellulase.

	Eaa)	*T* [K]	*K*_inact_ b)	Half‐lifec)	Δ*H* a)	Δ*G* a)	Δ*S* a)
Free cellulase	55.898	333.15	0.003455	200.6505	53.13	86.216	−0.09931
		343.15	0.006679	103.7847	53.047	87.008	−0.09897
		353.15	0.010824	64.0374	52.964	88.211	−0.09981
Cellulase@Cs/PVA/Ga	47.952	333.15	0.001382	501.6263	45.184	88.753	−0.13078
		343.15	0.002303	300.9758	45.101	90.045	−0.13097
		353.15	0.003685	188.1099	45.018	91.374	−0.13126

a) Activation energy (Ea), enthalpy change (Δ*H*), Gibbs free energy change (Δ*G*), and entropy change (Δ*S*). All values are in kJ mol^−^^1^;

b) First‐order inactivation rate constant expressed in min^−1^;

c) Half‐life expressed in minutes.

Comparing the enthalpy changes, free cellulase exhibited higher Δ*H* values than cellulase@Cs/PVA/Ga, implying greater conformational changes in the free enzyme during thermal inactivation. Both enzymes showed negative Δ*S* values, indicating conformational changes without aggregation during thermal inactivation; however, the differences in Δ*S* suggest that the structural flexibility of free cellulase has increased.

Our results demonstrated that both Δ*H* and Δ*S* for immobilized cellulase were lower than those for the free enzyme. This is consistent with previous finding,^[^ ^53^ ^]^ which indicate the structural rigidification occurs due to multipoint attachment. The immobilized enzyme adopts a more ordered structure that demands fewer enthalpic changes to reach the transition state. However, this enhanced stability comes at the cost of lower entropy. This combined effect leads to a higher Δ*G*, thereby increasing resistance to thermal inactivation and lowering sensitivity to temperature variations.^[^ ^53^ ^]^

In a study conducted by Yan et al., it was observed that the thermodynamic parameters of immobilized β‐glucosidase exhibited a notable decrease in Δ*H* compared to the free enzyme. This reduction may be linked to restricted enzyme flexibility and the formation of stabilizing interactions with the immobilization surface.^[^ ^54^ ^]^ Furthermore, research conducted by another team^[^ ^55^ ^]^ revealed a decline in the enzyme's Δ*H* due to immobilization, suggesting that fewer bond rearrangements are necessary to achieve the transition state. This lower enthalpic demand implies that immobilized enzymes require less energy for activation and exhibit improved resistance to temperature‐induced fluctuations.^[^ ^55^ ^]^

Overall, the assessment of Δ*G*, Δ*H*, and Δ*S* indicates that the connection to support with covalent bonding enhances the thermostability and reduces conformational changes in cellulase during heat inactivation.

Thermal inactivation was evaluated using the constant inactivation rate (*K*_inact_), reflecting enzyme stability. The first‐order kinetics inactivation model was derived from the semilogarithmic plot of remaining activity versus time to calculate the inactivation rate constant and half‐life. **Table** **3** presents the half‐life durations for both bare and immobilized enzymes. The half‐life of immobilized cellulase was significantly longer than that of free cellulase, consistent with previous research.^[^ ^44^ ^,^ ^49^ ^]^ This finding highlights the enzyme's stability and reduced inactivation with increased temperature exposure, supporting that chitosan microspheres may protect the enzyme from environmental factors.^[^ ^56^ ^]^ Glutaraldehyde, possessing two aldehyde groups, forms covalent bonds with the —NH_2_ groups present in both chitosan and the enzyme during immobilization. This reaction leads to the formation of Schiff base linkages that provide stable anchoring. The resulting cross‐linked structure enhances the enzyme's thermal stability by establishing a protective microenvironment and strengthening interactions between enzyme molecules and their support matrix. This stabilization, through multiple covalent linkages, limits conformational flexibility and prevents protein unfolding, making immobilized enzymes more resistant to denaturation than their free counterparts.

**Table 3:** Reusability of immobilized cellulase on chitosan supports.

Type of support	Number of cycles	Residual activity [%]	Reference
Polyvinyl alcohol‐coated chitosan beads	8	52	[44]
Magnetic chitosan nanoparticles	10	50	[59]
Magnetic chitosan microspheres	9	53.51	[67]
Fe_3_O_4_/GO/CS	8	49	[68]
SA‐PEG‐CS	5	59.42	[69]
Fe_3_O_4_‐GO‐CS nanocomposites	5	71	[49]
Chitosan functionalized magnetic nanoparticles	5	27.95	[70]
Cs/PVA/Ga	7a)	77	This study
	7b)	90	

a) continuous in 1 day;

b) noncontinuous in 7 days.

### 2.7 Reusability of Cellulase@Cs/PVA/Ga

**Figure 5:**
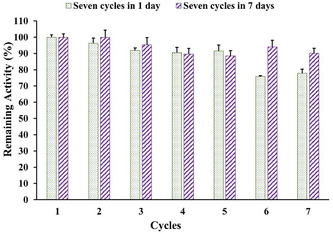
Reusability of cellulase@Cs/PVA/Ga after seven cycles in 1 and 7 days.

The effectiveness of the immobilized enzyme's catalytic activity was evaluated using repeated DNS assays to evaluate its reusability. The cellulase activity was measured several times and expressed as a residual activity of its initial activity (**Figure** **5**).

In the continuous method, cellulase@Cs/PVA/Ga activity decreased with each reuse, reaching 77% by the seventh cycle. In the noncontinuous method, the enzyme was separated via centrifugation after catalysis, washed with PBS, and stored at 4 °C overnight. After seven cycles, the residual activity of the immobilized cellulase on the Cs/PVA/Ga beads was 90%. The reusability of the covalently bonded cellulase on various chitosan supports is detailed in Table 3. It seems that the physical and chemical properties of the Cs/PVA/Ga matrix directly dictate cellulase stability and reusability. On the chemical level, covalent cross‐linking via C=N imine bonds and extensive hydrogen bonding (—OH and —NH groups) contribute to structural robustness, reduce enzyme leaching, and create a protective microenvironment. On the physical side, enhanced mechanical strength and minimized swelling help prevent deterioration of the support during repeated operations. These attributes ensure the enzyme remains catalytically active and functional over multiple reaction cycles.

Despite the use of covalent bonding in continuous and noncontinuous reuse methods, differences in residual enzyme activity were observed. According to a prior study,^[^ ^57^ ^]^ two main factors may contribute to immobilized enzyme inactivation: 1) enzyme loss during repeated handling, and 2) conformational changes or denaturation over successive cycles. In this study, both reuse methods included washing after each cycle to remove residual substrates or inhibitors. The key distinction was that the noncontinuous method incorporated an overnight storage step at 4 °C, allowing the enzyme's structure to refold. Previous research^[^ ^58^ ^]^ demonstrated that immobilized enzymes show enhanced stability under cold conditions, prolonging their half‐life. During continuous operations, noncovalent interactions can weaken, disrupting enzyme conformation. However, storage at 4 °C allows these interactions to reform, enabling the enzyme to recover its active structure.

### 2.8 Deinking Ability of the Free and Immobilized Enzyme

#### 2.8.1 Evaluation of Cellulose, Hemicellulose, and Lignin Content

**Figure 6:**
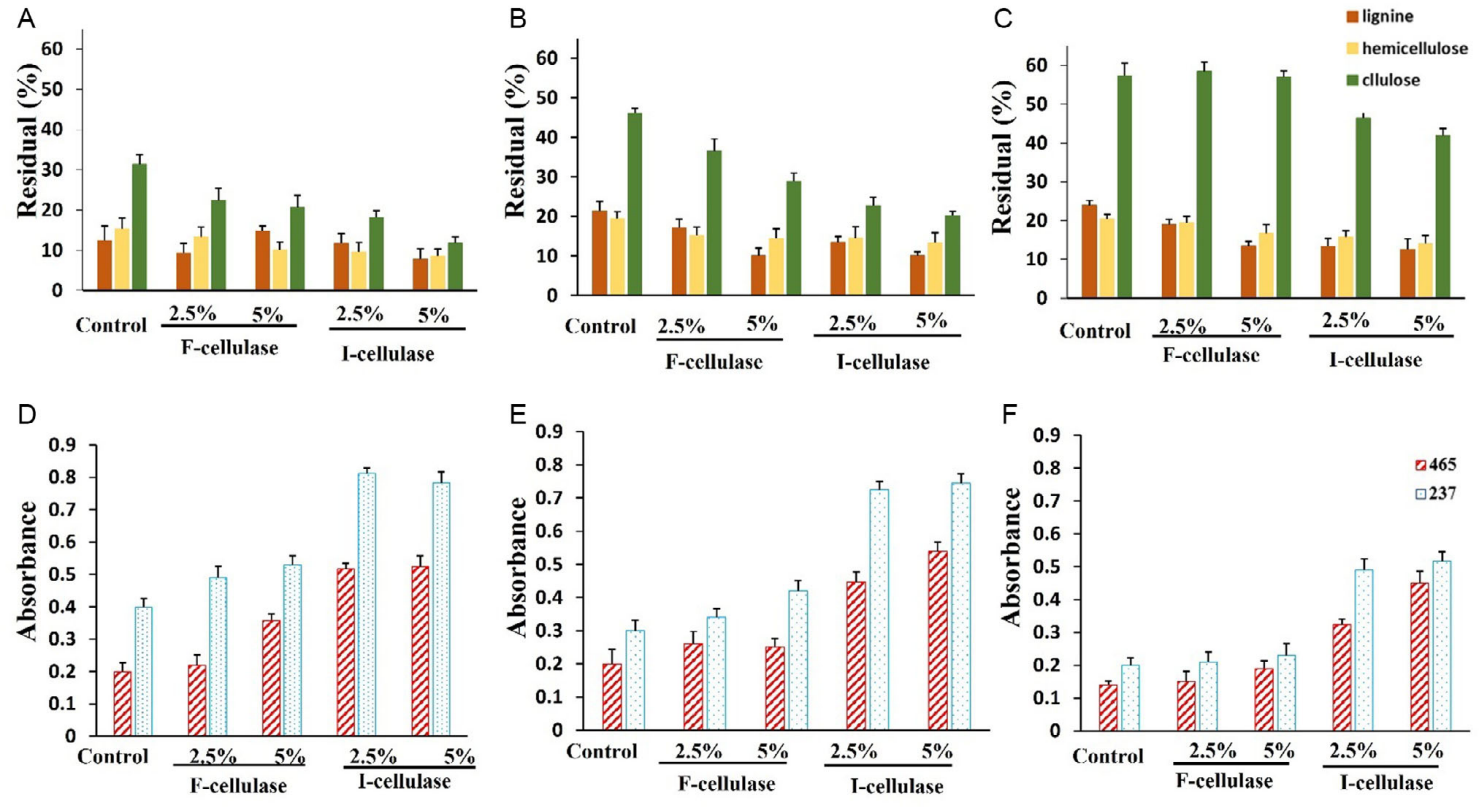
A–C) The residual contents of cellulose, hemicellulose, and lignin (measured in percentage) in recycled old newspaper pulp that was treated with free cellulase (F‐cellulase) and immobilized cellulase (I‐cellulase) at varying pulp consistencies. D–F) The absorbance values of hydrophobic compounds at 465 nm and phenolic compounds at 237 nm that were released into the effluent during the enzymatic deinking process at different pulp consistencies: (A,D) 3% (w/v), (B,E) 5% (w/v), and (C,F) 10% (w/v), using enzyme dosages of 2.5% and 5%.

Optimizing the consistency of recycled pulp and enzyme dosage is vital for effectively deinking old newspaper pulp. We assessed the cellulose, hemicellulose, and lignin content. Our findings demonstrated that at 3% (w/v) newspaper pulp consistency, increasing the free enzyme dosage from 2.5% to 5% significantly lowered cellulose and hemicellulose levels (**Figure** **6**A) due to enzyme‐mediated fiber degradation. Unlike free cellulase, cellulase@Cs/PVA/Ga showed enhanced activity, further reducing residual cellulose and hemicellulose. At 5% newspaper pulp consistency (Figure 6B), enzyme‐treated samples exhibited lower components than controls, with the most significant decrease seen with higher cellulase@Cs/PVA/Ga. However, the residual cellulose and hemicellulose were higher at 5% pulp than at 3% with the same enzyme dosage, indicating insufficient enzyme activity for effective fiber breakdown. Free enzymes showed no significant activity at 10% pulp (Figure 6C), whereas, cellulase@Cs/PVA/Ga still reduced cellulose and hemicellulose levels, indicating some fiber degradation.

**Figure 7:**
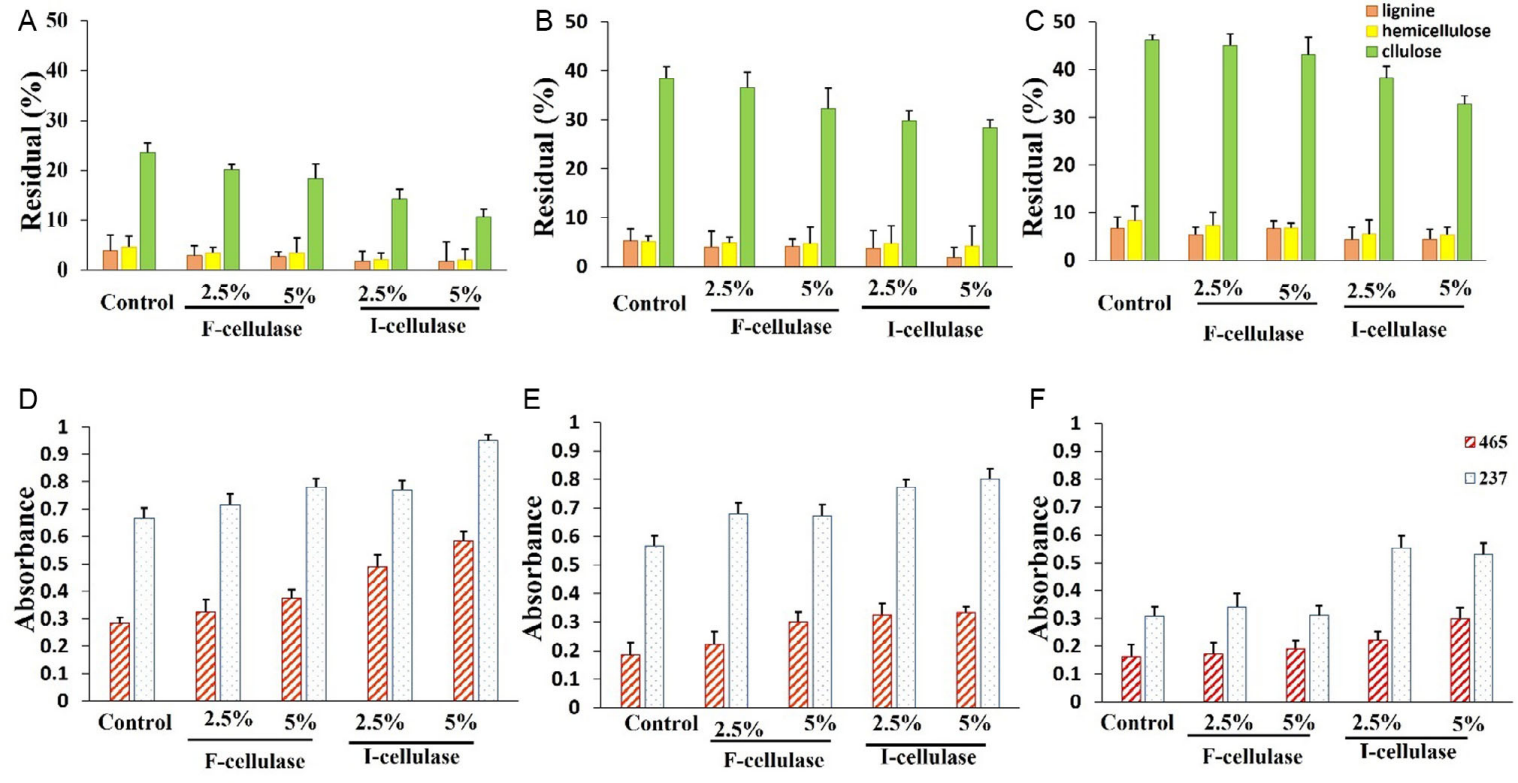
A–C) The residual contents of cellulose, hemicellulose, and lignin (measured in percentage) in recycled printed paper pulp that was treated with free cellulase (F‐cellulase) and immobilized cellulase (I‐cellulase) at varying pulp consistencies. D–F) The absorbance values of hydrophobic compounds at 465 nm and phenolic compounds at 237 nm that were released into the effluent during the enzymatic deinking process at different pulp consistencies: (A,D) 3% (w/v), (B,E) 5% (w/v), and (C,F) 10% (w/v), using enzyme dosages of 2.5% and 5%.

Results about printed paper showed that increasing free enzyme dosage improved fiber degradation at 3% pulp consistency (**Figure** **7**A). At the same time, immobilized cellulase (cellulase@Cs/PVA/Ga) was more effective, achieving the lowest residual cellulose and hemicellulose. At 5% consistency (Figure 7B), enzyme‐treated samples had lower levels than the control, but residual amounts were higher than at 3%. Free enzymes had little effect at 10% (Figure 7C), while cellulase@Cs/PVA/Ga still reduced cellulose and hemicellulose.

Overall, the most notable reduction of cellulose and hemicellulose occurred at 3% pulp consistency, aligning with previous research that indicated more significant reductions at lower pulp concentrations.^[^ ^3^ ^]^ The decline in residual cellulose and hemicellulose in recycled paper signifies decreased paper quality. Since free enzymes were ineffective at 10% pulp, treating 5% pulp with free enzymes is optimal for recycling, while immobilized enzymes successfully break down fibers at 10% pulp concentration.

#### 2.8.2 Evaluation of Hydrophobic and Aromatic Compounds in Effluent

This research investigated the release of hydrophobic and phenolic compounds in effluent during the enzymatic deinking process. The analysis showed that enzymatic activity increased the concentration of these ink‐related substances. Maximum release of hydrophobic and phenolic compounds occurred at 3% (w/v) pulp paper (Figure 6D), consistent with biodeinking efficiency findings.^[^ ^28^ ^,^ ^59^ ^]^ As pulp concentration rose, the levels of these compounds declined, with the lowest release at 10% pulp (Figure 6F). At this concentration, the release levels with free enzyme matched those of the control, indicating the enzyme's ineffectiveness. However, samples with 10% (w/v) pulp treated with cellulase@/Cs/PVA/Ga showed higher release than controls.

When the printed paper pulp consistency was at 3%, we observed the highest release of hydrophobic and phenolic compounds, especially with a more potent enzyme (5% cellulase@Cs/PVA/Ga) (Figure 7D), aligning with our findings that increasing the consistency of the enzyme improves deinking results. At 5% (w/v) printed pulp (Figure 7E), enzyme treatments still released more compounds than without enzymes. However, due to an increase in the pulp ratio to the enzyme concentration, the release of chemical compounds was reduced. At 10% consistency of printed paper pulp (Figure 7F), free enzymes did not improve compound release compared to the control, indicating reduced effectiveness. However, the immobilized enzyme continued to perform well, significantly enhancing the release of hydrophobic and phenolic compounds.

## 3 Conclusion

Cs/PVA/Ga beads are a good choice for recycling paper. The enzyme in these beads can handle high temperatures, which means it could be helpful for a long time in factories. In the future, we should look at the costs of using the enzyme when it is stuck to the beads compared to when it is free. The paper recycling strategy involves several stages and requires that white grades be effectively deinked to restore their optical qualities. Deinkability refers to the ability to remove most of the ink or toner from printed materials. Therefore, using ligninolytic enzymes can be beneficial in the paper recycling industry.

It is crucial to optimize the enzyme‐to‐paper ratio to ensure that recycled paper is high quality (containing a significant amount of cellulose and hemicellulose) and achieves effective deinking. Immobilized enzymes enhance cost‐effectiveness and improve performance, especially when they can be reused. This study achieved effective deinking of old newspapers using immobilized cellulase at a dosage of 5% and a pulp consistency of 10%. The enzymatic deinking method described here is more ecofriendly than conventional chemical techniques. The reduction in toxic effluents and improved recyclability of the enzyme contribute to sustainable waste management in the paper industry.

When assessing immobilized cellulase systems for industrial applications, evaluating several key performance metrics beyond residual activity is vital. The enzyme's catalytic efficiency, reflected in substrate conversion rates and product yield, is a primary indicator of system effectiveness. Additionally, tracking enzyme leaching is necessary to confirm its retention and structural stability during use. Evaluating the enzyme's operational half‐life across multiple cycles is especially important, as it directly reflects the reusability and overall cost‐effectiveness of the system. Moreover, the immobilized system must withstand the demanding conditions of industrial processes, including the presence of inhibitors and harsh reaction environments. The mechanical and structural durability of the support matrix must be sufficient to resist swelling, abrasion, and pressure variations throughout repeated use. These factors are essential for ensuring the system's long‐term functional stability and economic feasibility in large‐scale industrial applications. Further studies on the environmental impact of large‐scale implementation of this method could reinforce its role in green industrial processing.

## 4 Experimental Section

Imidazole, sulfuric acid, glutaraldehyde, sodium hydroxide, acetic acid solution, and chitosan were purchased from Merck Millipore. Isopropyl β‐D‐thiogalactoside (IPTG) and carboxymethylcellulose (CMC) were purchased from Sigma Aldrich. All the reagents were of analytical grade. Transformed bacterial strains containing the cellulase gene had been prepared in a previous study.^[^ ^60^ ^]^

4.1

4.1.1

##### Cellulase Expression and Purification

The transformed bacterial strain was induced with 1 mM IPTG at 20 °C to express recombinant cellulase. Cells were collected by centrifugation (4000 g for 10 min), resuspended in 5 ml of lysis buffer (50 mM Tris‐HCl, 17 g L^−^^1^ NaCl, 0.6 g L^−^^1^ imidazole, pH 7.8), and sonicated (13 cycles of 20 s pulse with a 30 s interval on ice). The supernatant from centrifuging crude cell extracts (10,000 × g for 30 min at 4 °C) was purified using Ni–NTA sepharose resin, following the previous method.^[^ ^61^ ^]^

##### Preparation of Chitosan/PVA/GA Cross‐Linked Cellulase

Cellulase@Cs/PVA/Ga was synthesized with modifications based on the method by Pawar et al.^[^ ^62^ ^]^ First, 0.1 g of chitosan was dissolved in 8 ml of 0.1% v/v acetic acid, kept at room temperature, and permitted to rest for 24 h. The pH was modified to 5.5 by incorporating 1 M NaOH, and the mixture was agitated at 4 °C for 1 h. Cross‐linking was initiated by stirring 2 ml of purified enzyme and 0.5% w/v glutaraldehyde into the chitosan solution for 6 h at 4 °C. Solution B was prepared by dissolving 0.05 g of PVA in 5 ml of distilled water heated to 80 °C and subsequently allowed to cool to room temperature.

Then, solution A was introduced dropwise into solution B while maintaining a temperature of 4 °C. The mixture was stirred for 3 h, aliquoted into microtubes, and stored at 4 °C for 24 h. Following storage, the tubes underwent centrifugation at 9000 rpm for 4 min at a temperature of 4 °C, and the supernatant was utilized to determine the concentration of free enzyme. The immobilized cellulase, termed cellulase@Cs/PVA/Ga, was stored at −20 °C for further analysis. Immobilization efficiency (%) was calculated using the (Equation 1).^[^ ^63^ ^]^

(1) Immobilization efficiency(%)=((Ci−Cs) )/(Ci)× 100

where (Ci) is the protein concentration before immobilization and (Cs) is the concentration after immobilization in the supernatant.

##### Assay of Cellulase Activity

All enzyme activity measurements assumed equal concentrations of free and immobilized enzymes. With an enzyme immobilization efficiency of 92%, 0.1 mg of cellulase@Cs/PVA/GA equates to 0.02 mg of the enzyme. Enzyme activity was assessed using identical free and immobilized cellulase concentrations in 60 μL of 1% (w/v) CMC at pH 5. Reducing sugars were measured using the dinitrosalicylic acid (DNS) method.^[^ ^64^ ^]^

##### Determination of Optimum Temperature

To determine the optimal temperature, 60 μL of CMC was incubated at 30–80 °C. Subsequently, 0.02 mg mL^−^^1^ of free enzyme and 0.1 mg mL^−^^1^ of cellulase@Cs/PVA/Ga were added to the reaction. After 30 min, the reaction was stopped by adding 120 μL of DNS solution and boiling for 5 min. The mixture was spun in a centrifuge at 11,000 rpm for 5 min to separate the cellulase@Cs/PVA/Ga beads, and the activities of both free cellulase and cellulase@Cs/PVA/Ga were measured by assessing the optical density at 540 nm.

##### Determination of Optimum pH

Various buffer solutions were prepared: citrate buffer (50 mM) at pH 4 and 5, phosphate buffer (50 mM) at pH 7 and 8, and carbonate‐bicarbonate buffer (50 mM) at pH 9. 1% (w/v) CMC solution was prepared in each buffer. Then, 0.02 mg mL^−1^ of free cellulase and 0.1 mg mL^−1^ of cellulase@Cs/PVA/Ga were added to 60 μL of the CMC solution at the respective pH levels. The mixtures were incubated for 30 min at their optimal temperatures. Following incubation, 120 μL of DNS solution was added, and the mixtures were boiled for 5 min to stop the reaction. The enzyme activity was evaluated by quantifying the amount of reducing sugars released at 540 nm.

##### Kinetic Parameters Calculation

The kinetic parameters of free cellulase and cellulase@Cs/PVA/Ga were analyzed using a Hanes–Woolf plot with carboxymethyl cellulose substrates at concentrations of 0.1, 0.3, 0.6, 1, 2, and 2.5 mg mL^−1^. The substrates were prepared in a buffer at the optimal pH for free and immobilized enzymes, and reactions were conducted at each enzyme's optimal temperature. After DNS addition and boiling, samples were centrifuged to isolate the chitosan beads, and the supernatant absorbance was measured at 540 nm.

##### Assessment of Thermostability and Storage Stability

To assess the thermostability of cellulase and the cellulase@Cs/PVA/Ga, the enzymes were incubated at 60, 70, and 8 0°C. At intervals of 30 and 60 min, samples were cooled on ice for 10 min. After cooling, 60 µl of substrate was added to each sample and incubated at the optimal temperature for 30 min. The remaining enzyme activity was then measured to evaluate the effects of heat treatment. The activity of both the free and immobilized enzymes was considered 100% prior to thermal treatment. The storage stability of free and immobilized enzymes was evaluated over 14 days at 4 °C, with evaluations occurring daily. The activity of both the free and immobilized enzymes was considered 100% on the first day.

##### Thermodynamic Parameters

0.1 mg of cellulase@Cs/PVA/Ga and 0.02 mg mL^−1^ of free cellulase (equal concentrations of free and immobilized enzymes) were placed in test tubes containing PBS at the optimal pH and incubated in a water bath at 60, 70, and 80 °C for 1.5 h. The supernatant was collected every 30 min after cooling the cellulase@Cs/PVA/Ga tubes on ice. CMC was then added to both test tubes (free and immobilized enzymes), which were maintained at optimal temperature for an additional 30 min. Residual activity was measured by adding DNS to the reaction tubes and calculating the glucose released at 540 nm. The semi‐log plot of residual activity was used to determine the inactivation rate constant (*k*_inact_) by plotting against time. The half‐life of the enzymes and the Arrhenius plot were also investigated.

The Arrhenius plot was created by graphing the natural logarithm of *k*_inact_ against the reciprocal of absolute temperatures, as shown in (Equation 2).

(2) Ln(k)=Ln(c)−(Ea/RT)

In this equation, *R* is the universal gas constant (8.31 J mol^−1 ^K^−1^), Ln(c) is the y‐intercept, *T* is the absolute temperature in Kelvin (K), and Ea represents the activation energy of thermal inactivation (J mol^−1^).

The enthalpy of inactivation (Δ*H*) and Gibb's free energy (Δ*G*) were calculated by (Equation 3) – (4).

(3) ΔH=Ea−RT

(4) ΔG=−RT.Ln(kh/KBT)

where *h* is the Planck's constant (6.6262 × 10^−34 ^Js), *k*B is the Boltzmann's constant (1.3806 × 10^−23^ J K^−1^), k (s^−1^), and *T* is the absolute temperature (K). The entropy can be calculated using the (Equation 5).

(5) ΔS=(ΔH−ΔG)/T

##### Reusability of Cellulase@Cs/PVA/Ga

The cellulase@Cs/PVA/Ga reusability was evaluated over seven cycles, using continuous and noncontinuous processes. In the continuous system, after each 30 min reaction at 60 °C with 60 μL CMC (pH 8), the supernatant was collected via centrifugation (9000 rpm for 5 min), and enzyme activity was measured at 540 nm following boiling with DNS solution. The cellulase@Cs/PVA/Ga was washed with 50 mM phosphate buffer (pH 7), and the substrate was reintroduced for subsequent activity measurements.

In the noncontinuous procedure, after each reaction, the cellulase@Cs/PVA/Ga beads were rinsed with 50 mM phosphate buffer (pH 7) and stored at 4 °C overnight. The enzyme activity was assessed the next day by adding the substrate.

##### Enzymatic Deinking of Newspaper Waste

The old newspaper was cut into 2 cm pieces and soaked in distilled water for 2 h. After filtering and washing, the papers were dried in an oven at 40 °C for 20 h. This dried paper pulp was then used for cellulase‐based enzymatic deinking.^[^ ^65^ ^]^ The paper was resuspended in 50 mM phosphate buffer at pH 6 to create pulp consistencies of 3%, 5%, and 10% (w/v). Free and immobilized cellulase was added at a dosage of 2.5% and 5% (w/w).

The pulp was incubated with cellulase and cellulase@Cs/PVA/Ga for 2 h at 55 °C, followed by heat inactivation in a boiling water bath for 5 min. The treated pulp was filtered to obtain an effluent, which was analyzed for hydrophobic and phenolic compounds released during deinking. Absorption of these compounds was measured at 465 and 237 nm.^[^ ^3^ ^]^ In a related experiment, the deinking effect of both free and immobilized enzymes was examined on printed papers with the HP Color LaserJet 5550 Printer. For both test series, a control sample without any enzyme was included. The experiment was conducted in triplicate.

##### Assessment of Cellulose, Hemicellulose, and Lignin Content

The treated papers were dried at 50 °C for 3 h and boiled three times in 70% ethanol for 15 min each. After cooling, the samples were centrifuged at 5000 rpm to remove the ethanol. The paper pulp was washed with distilled water and dried at 40 °C.

The weight of the dried pulp was measured and divided into two equal parts, A1 and A2. 24% v/v KOH solution was added to A1 and incubated for 4 h at 25 °C. The papers were washed with distilled water and dried at 80 °C for 16 h, resulting in group B.

A2 was treated with 72% v/v H_2_SO_4_ and left at room temperature for 3 h. The samples were diluted to a final 5% v/v H_2_SO_4_ concentration and refluxed.^[^ ^66^ ^]^ To remove H_2_SO_4_ completely, the samples were washed with distilled water, centrifugated at 5000 rpm for 5 mins, then dried at 80 °C for 16 h, resulting in group C.

The residual amounts of cellulose, hemicellulose, and lignin in the treated pulps were determined using specific formulas.^[^ ^66^ ^]^

(6) Cellulose=B−C

(7) Hemicellulose=A1−B

(8) Lignin=C

## Conflict of Interest

The authors declare no conflict of interest.

## Data Availability

The data that support the findings of this study are available from the corresponding author upon reasonable request.
